# Global health-based virtual exchange to improve intercultural competency in students: Long-lasting impacts and areas for improvement

**DOI:** 10.3389/fpubh.2022.1044487

**Published:** 2022-11-14

**Authors:** Stuart J. Case, Sarah L. Collins, Elizabeth A. Wood

**Affiliations:** ^1^College of Public Health and Health Professions, University of Florida, Gainesville, FL, United States; ^2^Eck Institute for Global Health, University of Notre Dame, Notre Dame, IN, United States

**Keywords:** virtual exchange, transformative learning theory, directed content analysis, global health, post-secondary education, intercultural competency

## Abstract

**Introduction:**

As public health expands its role in global settings, the need to develop intercultural competency for public health students also grows. One initiative being applied to promote global awareness is the use of virtual exchange (VE) programs. VE programs promote collaborative online international learning (COIL) and allow students from different countries to connect and work together on projects related to their field of study; however, there is little research around the long-term impacts of these programs.

**Methods:**

Undergraduate pre-health students from the United States who participated in a VE program a year prior were interviewed about their experiences engaging with undergraduate medical students in Egypt. They were asked if the experience impacted their current behaviors, skills, or knowledge, and what improvements could be made to the program. Mezirow's Transformative Learning Theory (TLT) served as the theoretical framework, grounding interview instrument development and directed content analysis procedures. Researchers also engaged in inductive analysis to capture other salient themes.

**Results:**

Ten students were interviewed with a majority engaging in either of the two final stages of Mezirow's TLT: “building of self-confidence and self-competence” (60%) and “reintegration” (50%). Other salient themes found were intercultural interactions, VE appreciation, and VE improvements. When describing their experience in one word, students overwhelmingly provided words with positive connotations (80%), with the negative responses being explained by the structure and presentation of the VE.

**Discussion:**

Students were able to apply lessons they learned during the VE within a 1-year follow-up period. This is beneficial, as health professionals require intercultural competency to promote and provide improved health outcomes. Results from this study indicate the need for structure when conducting a VE, addressing the need to increase the number of direct interactions and thereby promoting more intercultural exchanges. Likewise, the interviews demonstrated that changes in course instruction need to be implemented gradually to allow for students to adjust to unfamiliar teaching methods.

## Introduction

The increased diversification of the United States along with the expanding role of public health in a global setting has brought awareness to the pervasive nature of intercultural interactions within the field (1, 2). The need for intercultural competency within public health is immense, as public health professionals often work in diverse population- and community-based settings (3). In addition, certain subpopulations with diverse and intersecting sociodemographic identities tend to have worse health outcomes for specific conditions (4, 5), creating the need for heightened cultural awareness and humility among public health professionals. As defined in Deardorff's Intercultural Competence Framework, intercultural competency is the capacity to develop skills and attitudes that enable individuals to behave and communicate appropriately in intercultural interactions (6). Intercultural competency, as described by Bennett (7), posits that there are cognitive and behavioral skills that influence interactions with people from other cultural backgrounds. Intercultural competency is often the bedrock of understanding one's own paradigmatic assumptions about the world (8), thus making it a relevant practice within higher education. As such, it should be a priority among institutions of higher education to develop these skills within public health students.

While initiatives such as study abroad programs have been traditionally used to promote intercultural exchanges (9), this approach can be inequitable, with fewer than 10% of US students being able to participate and an overwhelming majority identifying as non-Hispanic, Caucasians (10). To encourage the development of intercultural competency in post-secondary students, collaborative online international learning (COIL) has been implemented to foster connections between students in different countries and create global learning networks (11, 12). Assisted by recent innovations in video conferencing and telecollaborations (13), the capacity for schools to carry out COIL-based programs has expanded. One approach used to promote collaborative, international learning at post-secondary institutions is through virtual exchange (VE) programs. Within VE, students in different countries can engage in telecollaboration to exchange their ideas, opinions, and backgrounds in a learning environment (14).

### Mezirow's Transformative Learning Theory

Mezirow's Transformative Learning Theory (TLT) consists of 10 phases addressing how an individual's ability to learn is dependent upon their ability to change their frame of reference and assumptions, allowing them to be more open to new ideas and information (15). With the TLT, learning is believed to transpire through a stage-based process, beginning with an individual questioning their assumptions through a disorienting dilemma and concluding with the final two phases of the TLT, “Building of Self-Competence and Self-Confidence” and “Reintegration,” (15). Specifically, the last two phases address the ability of students to apply their experiences beyond the classroom setting in society, with the former inquiring about the execution of new roles and behaviors while the latter focuses on making these new behaviors habitual (15). Mezirow postulates that learning is most effective when there is an active dialogue, equal opportunities to participate, and reflection throughout, thereby permitting the individual to alter their normative beliefs (16). One area of learning which the TLT can be applied to is intercultural exchanges. Oftentimes, students' perceived stereotypes of others can limit their ability to immerse themselves within their program (17). Despite this, Mezirow argues that “through critical reflection, we become emancipated from communication that is distorted by cultural constraints,” such as one's preconceptions of another culture (16).

While VE programs have been carried out to promote intercultural learning among health-based students (18–20), there is limited research regarding the long-term impacts these programs have on students. It is necessary to determine whether these programs are able to maintain enduring impacts on students to truly determine the effectiveness of VE as a pedagogical strategy. Moreover, there is limited literature on the capability of COIL-based educational programs in pre-health and public health fields (20–22), creating a need to conduct further research on how VEs should be programmed to best serve these student populations.

### Study aims

The goal of this qualitative evaluation study was to examine pre-health students' perspectives on their COIL-based, VE experience and determine the long-term impacts, if any, on the students' intercultural competency. For the purpose of this study, long-lasting impacts were defined by explicit displays of knowledge gained from the VE by the students, 1 year after the initial VE. In addition, this study explored program implementation, participant satisfaction, challenges, and barriers faced by students during the VE program. Provided with the work of Wood et al. (20), it has been demonstrated that VE programs can facilitate a majority of the stages within the TLT, but they were unable to determine students' capability in demonstrating the final two stages related to integrating the theoretical into practice. This study seeks to understand whether VE programs can result in students progressing to and through the final two stages of the TLT: “Building of Self-Competence and Self-Confidence” and “Reintegration.”

## Methods

### Data collection

In the initial VE, 108 undergraduate pre-health students at a large, public university in the Southeast region of the United States enrolled in a global public health course collaborated with 32 undergraduate medical students studying at a university in Egypt during the Spring 2021 semester. Students from the US and Egypt were placed into groups with one another and communicated with each other in English *via* text or Zoom (20). The VE lasted for 5 weeks, with the content each week focusing on different public health issues, such as COVID-19, and how the approach to these issues differs based on students' home countries and the perspectives that exist within them (20). The US Students were required to complete weekly critical reflection-based assignments, a collaborative presentation with the Egyptian students on global health threats, and a summative paper, prompting US students to reflect on their experiences with the Egyptian students (20).

In the Spring of 2022, a year following the VE, the original institutional review board (IRB) study was updated and revised to include follow-up interviews of the US students who had participated in the VE. The IRB revision process was completed and approved in February 2022 (IRB#202003293). Following IRB approval, students who had participated in the virtual exchange in 2021 were contacted through their university-affiliated email, requesting their participation in an interview describing their experiences post-VE. Interviews were facilitated by one study researcher and conducted virtually *via* Zoom in a semi-structured format (23). The interview guide was developed based on Mezirow's final two TLT stages, as well as formative and summative evaluation questions of the program (Appendix A). The instrument was vetted among all of the investigators, providing a source of technical and critical feedback to ensure the guide's validity (24). Additional probing questions were asked to elicit further opinions and suggestions from the students. These probing questions were not structured, as the researchers hoped to have students express their genuine experiences, rather than shape responses through pre-planned probes, in accordance with semi-structured interviewing protocol (25). Interviews were audio-recorded and transcribed using Otter.AI software. Transcripts were reviewed for quality assurance by two researchers.

### Data analysis

Two researchers were utilized to independently analyze the data, providing a source of investigator triangulation (26). The researchers used both inductive and directed content analysis approaches to capture salient themes using NVivo software (27–29). The last two stages of Mezirow's TLT were defined a priori to guide the analysis deductively; however, inductive coding was used to capture themes that were outside the scope of Mezirow's TLT, but still offered rich insight into the VE program. After an initial review, the researchers convened to find consensus on overall themes, subthemes, and their respective operational definitions. Once consensus was met, the researchers conducted a second round of independent deductive analysis using the established codebook. Following the second round of independent analysis, the researchers convened until consensus was met.

## Results

Ten US students who partook in the 2021 VE participated in the interviews. The sample included four students who identify as male and six who identify as female. Half of the students interviewed were either born outside of the United States or were first-generation Americans. Interviews lasted between 10 and 30 min. Three themes, Intercultural Interactions, VE Appreciation, and VE Improvement, were identified in addition to the a priori themes. Six subthemes were also identified: Intercultural Interactions prior to VE, Intercultural Interactions during the VE, VE Appreciation related to modes of communication, VE Appreciation related to its application to future health professions, VE Improvements related to design, and VE Improvements related to logistics. Several accounts of the a priori Mezirow themes were also recognized, resulting in the two predetermined themes: Building of Self-Competence and Self-Confidence, and Reintegration.

### Building of self-competence and self-confidence

The building of self-competence and self-confidence refers to a continued execution of new roles and involves an explicit mention beyond the classroom context, practicing relevant behaviors on a routine basis, which in turn, increases self-efficacy within new assumptions. Six out of ten participants believed the lessons they learned from the VE would directly translate to their future careers in health-related fields, leaving them more assured of their skills.

(Participant 6: Female, first-generation) This virtual exchange made me understand the importance of cultural humility…I think it is so important to consistently, proactively learn about what you're doing with other people, especially if it's people that you are unfamiliar with, even in the slightest. I think it saves a lot of stress, I think it shows that you have a level of respect for them, that you are willing to learn and adapt and work with them. And I think it just makes me a better student, and hopefully a better health practitioner in the future, that I can accept that I don't know everything. And I'm going to always want to learn and this virtual exchange just kind of put that into practice and solidified it.

### Reintegration

A demonstration of reintegration involves an expression that the refined assumption is now perceived as habitual, routine, and easy (i.e., without precontemplation) within one's everyday life. Five of the students had made efforts to reintegrate what they learned during the VE with their work. Students describe similar experiences integrating lessons from the VE in a variety of settings including international travels, online social interactions, as well as everyday settings.

(Participant 1: Female, American descent) In South Africa, I actually worked with a couple individuals who were from Egypt and I think it really helped me foster those interactions in the first place. [If] I hadn't gone through the virtual exchange, I would have probably still had those preconceived notions going into my work, and interacting with those individuals from Egypt and, you know, a ton of other places. But especially talking to the Egyptian students [in the spring] and knowing that I was going to be going to South Africa in the summer, it really helped, you know, okay, this isn't as foreign as I probably been led to think in the past.

### Intercultural interactions

Intercultural interactions involved any interactions held by students with people from another culture, locally or globally. These interactions were divided into those which occurred prior to the VE and those which happened during the VE; those after the VE were included in the themes regarding the TLM stages.

#### Prior to VE

Student responses were coded as prior to VE if there was any mention of experiences that involved an interaction between individuals from different cultural backgrounds that occurred prior to the virtual exchange (*n* = 10). These interactions could be intentional, such as traveling abroad, or more passive, such as being born outside the US or a first-generation American.

(Participant 6: Female, first-generation) Before 2020, when the pandemic hit, I actually used to be a very avid traveler…So I would say that, in terms of like being culturally aware, obviously, I don't know every single thing about every culture, but I do really make it a point to constantly learn and like understand and adapt, especially when I'm going to places that I have no idea what the customs and things of that nature are nearby.

Some students also talked about how either being a first-generation student or an immigrant to the United States impacts how they interact with people from other cultures.

(Participant 8: Female, first-generation) I would say that I'm more comfortable or more familiar with people that have experiences outside of the US or like, that have grown up in a different country, just because that's kind of what my parents' experience looked like. And so, because of that, I ask more questions. I want to know more about their experiences and see what it looks like to have now been in America for a little bit of time, if they're first gen, or if they're an immigrant and see how it compares to their previous experiences.

#### During the VE

Responses were coded as During the VE if they detailed interactions that occurred during the virtual exchange where the students from UF and Ain Shams University shared their backgrounds, cultures, experiences, or opinions. All of the participants described their intercultural experiences, however, many UF students shared discussions they had with the students from Egypt regarding the COVID-19 pandemic.

(Participant 2: Male, American descent) I think definitely, obviously, with the time period that the virtual exchange took place in, we focused a lot on COVID, and things like that. And it was very interesting to be able to compare a US experience to what is going on in Egypt, which, obviously, there were differences there and being able to see those differences. And, seeing a different perspective is a lot different than reading about it in a textbook or a news article or something like that.

There was also a collective astonishment among the UF students at how similar they were to the students from Egypt.

(Participant 3: Male, international-born) I just had the mindset that they would be, I don't know, they would just talk a different way. And like, I don't know, it's really interesting to just realize that, in the end, we're all pretty similar no matter what our background is, our religion, whatever. Like, we're all pretty similar. So I thought that was kind of interesting, and fun to see how we're pretty similar. And just the way we talk about lives, our lives and like, what we may have been going through school, stuff like that. I just thought that was really interesting.

### VE appreciation

The students were asked about what aspects of the VE they enjoyed. Any objective expression of how an individual personally benefited from the virtual exchange or any statements claiming the virtual exchange could result in benefits for others were coded as VE appreciation. Sixty percent (*n* = 6) of the students appreciated having the ability to meet with students from completely different backgrounds through the VE.

(Participant 2: Male, American descent) I think the entire concept of it is very interesting. It's very interesting to be able to meet up with students that are studying the same thing or something similar. They were medical students from Egypt, if I remember correctly, so from a totally different area of the world. And it was really interesting to just get that perspective on issues from something that is not our own. We take a lot of the same classes, especially in a smaller program, like public health with the same people. And not that we don't have a diverse public health program or something like that. But, you know, we all are like living in Gainesville, Florida right now for the most part. So it's different to hear something that is different.

Based on the responses provided, the theme was further divided into the subthemes of Modes of Communication and Application to Future Profession.

#### Modes of communication

This subtheme centered around the communication tools and technologies that were utilized during the VE. The students often used WhatsApp as their method of communication, providing a more informal way to communicate. Four of the participants specifically describe this as a benefit to the program.

(Participant 5: Female, American descent) I love the WhatsApp. I think we were all required to have some sort of communication. Some people did like Facebook groups or emails, I'm not really sure about the other groups, but we all got each other's Facebook information, and then made a WhatsApp and it was just I don't know, it was really fun to talk to them. And like where it felt like I was talking to a friend from home or like using emojis, like talking about school, studying for exams. So that was fun, like, just a mix up from the people you talk to every day, and they were so excited to do it. And so were we. So it felt less like an assignment and more like getting to know people.

#### Application to future health profession

This subtheme included discussion on how experiences during the exchange could be applied to future career settings in health-based areas. All (*n* = 10) of the students hoped to work in healthcare in the future and often talked about the variety of individuals they will work with in the future.

(Participant 3: Male, international-born) I'm going to OT school starting fall. So as someone who's going to be working in healthcare, I'm going to come across all types of people. Like I can come across anyone. So I think just being able to be patient when talking to someone. We didn't really have conversations the way we interacted, we talked through WhatsApp, we texted, but just in general, being able to be patient and understanding that not everyone is good with English and able to be understanding and patient enough to communicate with that person.

### VE improvements

In addition to questions about the aspects of the VE which the students enjoyed, they were also asked how they believe the VE could be improved. This theme involves any suggestions or criticisms made by the students with the purpose of addressing any aspect of the virtual exchange which should be changed, improved upon, or removed if a similar virtual exchange is conducted in the future. The areas of improvement were mainly pertaining to either the design of the VE or the logistics of the VE.

#### VE design

Critiques of the VE's design relates to any comments made regarding how the virtual exchange was introduced and implemented. To initiate the VE, an instructor at UF created a presentation to give an overview of what the VE would entail and what expectations should be had when interacting with the Egyptian students. Three of the students believed that the VE should have been introduced in a different manner.

(Participant 1: Female, American descent) I think it would be really beneficial and really cool to, kind of set the tone for the entire virtual exchange if maybe that very beginning part where we introduced ourselves to each other if people we are able to…record yourself and be like, ‘hello, I'm [name].' You know, I actually like talking to a camera and uploading that rather than sending a text message, I think that could definitely help to set the stage of the virtual exchange. So you know.... I think it would just make more of a lasting impression and foster that, you know, the connection piece a bit more.

Another area of improvement the UF students mentioned was in regard to the frequency and structure of communication with their Egyptian counterparts. The VE was set up with relatively relaxed requirements, due to expected difficulties with the time zones, but many students desired more structure.

(Participant 5: Female, American descent) …It was pretty much up to the group members, whether or not they wanted to participate and talk to people in the group. And since we independently made our communication, it wasn't really regulated. All we had to do was prove that we had talked to them at some point in the week. If I'm remembering right, I just think it would be good maybe for it to be going on a little longer or have more structured conversations, maybe like a zoom with everybody.

#### VE logistics

Half of the participants described improving the logistics of the VE regarding the coordination of connecting the groups of students, whether it be due to the time difference or other issues. Between the two universities there is a 6-h time difference, and this was frequently discussed as a barrier to communication.

(Participant 5: Female, American descent) Because the hours that we could talk to them, it's like, my guess would be their study hours, dinner, sleeping. So sometimes we would send a message and not get a response for a long time. And then by the time we got the response, we were, you know, just now waking up or sleeping, or in class or something. So the time difference at times was bad.

## Discussion

This analysis is among the few to examine whether a COIL-based VE program sustains long-lasting impacts on the behaviors of the students involved. Our findings point to several areas where VE programs can be improved upon as well as some of the limitations of Mezirow's TLT as a foundational theory. While students who were interviewed for this study did describe instances during the VE that were impactful or effective, these points were not included in the findings of this study as they were reinforcing the salient themes captured in Collins et al. (22). Here we expatiate our analysis to consider the larger, complex issues surrounding VE programs.

While most of the students were able to illustrate engaging in at least one of the two final stages of Mezirow's TLT, there were individuals who believed that the VE was not sufficient in promoting behavior change related to intercultural competency. One common explanation was US students' desire for more structure in when and how frequently to meet with the students from Egypt. The initial VE was purposefully structured to be somewhat fluid in its communication requirements and the number of interactions, as it has been demonstrated that, generally, moderately-structured instruction performs better than extremely lax or strict instruction (30). Despite this, the US students believed that given their unfamiliarity of the Egyptian students' background and the VE program, there should have been more requirements for interactions embedded within the VE. This experience is not isolated, as similar sentiments are felt by students studying abroad where time is required to acclimate to the new culture they are being immersed in (31).

These concerns of unfamiliarity, creating the aspiration for more structure, can be seen as unintended consequences of what Mezirow refers to as the “disorienting dilemma” (32). The disorienting dilemma, the first stage of the TLT, is an instance in which new information is brought to an individual or someone is put in a new situation, thereby encouraging them to engage in “perspective transformation” (32). Within the context of this VE, the disorienting dilemma was thought to be solely the interactions between American and Egyptian students; however, it is also likely that the dilemma can be attributed to the sudden change in course instruction caused by the VE. As such, the instructor can support transformative learning by isolating the disorienting dilemma to its desired source (33).

A possible solution, discussed by participants, to address this unfamiliarity of the VE would be to have the US students and the Egyptian students introduce themselves, as opposed to it being done by their respective instructors. Many US students expressed that they had preconceived conceptions regarding the Egyptian students which could have been ensconced through a short video blog (vlog), in which the students would show their daily activities and aspects of their culture. These blog-style videos have demonstrated to be effective in introducing new information in a classroom setting (34). Moreover, it has been shown that face-to-face interactions are preferable in intercultural exchanges when compared to the text-based introductions that most of the students in the VE employed (35). Given the benefits, the students would be able to represent their own culture and provide an authentic insight into their lives, thereby removing the sense of otherness.

While there are concerns regarding how the structure and presentation of the VE impact its ability to encourage transformative learning, the framework behind the TLT can also explain why the VE was unsuccessful in promoting long-lasting behavioral changes for some students. Interestingly, students who noted having less success in fulfilling the final two stages of the TLT typically identified as male. Students who identified as females were much more likely to say the VE had a more substantial impact and provide examples of how they have applied lessons from the VE. Likewise, the TLT's framework was based on a program to encourage women's re-entry programs in community college (32). Arguments have been made within the study abroad domain that females display higher intercultural activity than their male counterparts due to their ability to acknowledge and value differences among different cultures (36). In a recent study reviewing study abroad participation post-COVID pandemic, the gap between men and women has either widened or remained the same. However, it was speculated that the subject of study and the available study abroad programs tethered to those subjects play an important role (37). Consequently, given the TLT's inherent bias toward women, as well as a lack of focus on gender-based differences in adult education research and engagement (38), more efforts need to be directed to ensure that the TLT results in equitable outcomes for all genders. In doing so, VE programs could be modified in the future to increase the probability that *all* individuals sustain behavior change.

Another non-structural factor which impacted students' tendency to engage in either building of self-competence and self-confidence or reintegration was one's background. US students who expressed having backgrounds involving minimal intercultural exchange were more likely to express long-lasting behavior changes when compared to those who either immigrated to the United States or are first-generation Americans. Similar findings were found with a VE program with medical students from Australia and Indonesia, as students who previously had intercultural interactions with peers were less likely to experience benefits from the VE (18). Study abroad programs have utilized Eccles' value-expectancy theory to investigate how one's motivation within their study abroad program is related to how valuable the experience will be for them personally (39). With the VE program, students who had limited interactions with people from other cultures were able to benefit more from this unfamiliar situation while those who had a more international background already had developed intercultural competency and thereby did not see as much value in the experience.

## Strengths and limitations

There are several strengths and limitations that should be considered when interpreting our findings. Though our study had an adequate sample size to attain saturation (40), it was obtained through convenience sampling procedures. Due to the nature of convenience sampling, participants who had a particular interest in the VE, traditionally those with extreme perceptions (positive or negative) were more likely to participate. This likely introduced sampling bias to our study and limits the generalizability of the results. One strength of this study is the use of two researchers during the data analysis stage, which increases the validity of results through internal triangulation (26). Having two researchers reduces the influence of researcher bias and maintains the integrity of the data (41). Other efforts were made to increase the study's validity such as utilizing qualitative computer software for analysis and having one consistent interview facilitator (27).

## Conclusion

This study was one of the first to investigate the long-term integration of Mezirow's TLT, as well as evaluate a VE program within a global public health setting. In doing so, our findings suggest that the TLT may be biased toward women's transformative learning processes, adding to the critiques present toward this framework as an adult learning theory (22). Further research is warranted to assess the appropriateness of this theory among diverse student groups and learning environments, as higher education and public health continue to diversify as they transcend national settings and enter global spaces. Furthermore, this study evaluated the programmatic components of a VE program that were successful and can be improved upon. The insights provided by the participants allow for educators to adapt their pedagogical and instructional strategies in real time, thus allowing for an optimal VE program experience. However, quality assurance measures should always be taken, as VE experiences may change according to socioenvironmental and sociopolitical contexts.

## Data availability statement

The original contributions presented in the study are included in the article/Supplementary material, further inquiries can be directed to the corresponding author.

## Ethics statement

The studies involving human participants were reviewed and approved by University of Florida Institutional Review Board IRB#202003293. Written informed consent for participation was not required for this study in accordance with the national legislation and the institutional requirements.

## Author contributions

SCa and EW conceptualized the study design and methodologies, coded the data, and then analyzed the data for themes. SCo contributed to discussions on pedagogical frameworks as well as data analysis procedures and wrote the strengths and limitations as well as the conclusion. SCa collected data, wrote the introduction, results, and conclusion. EW wrote the methods. All authors engaged in editing the manuscript. All authors contributed to the article and approved the submitted version.

## Conflict of interest

The authors declare that the research was conducted in the absence of any commercial or financial relationships that could be construed as a potential conflict of interest.

## Publisher's note

All claims expressed in this article are solely those of the authors and do not necessarily represent those of their affiliated organizations, or those of the publisher, the editors and the reviewers. Any product that may be evaluated in this article, or claim that may be made by its manufacturer, is not guaranteed or endorsed by the publisher.

## References

[1] Lee B Martin M Matthews S Farrell C. State-level changes in US racial and ethnic diversity, 1980 to 2015: a universal trend? Demogr Res. (2017) 37:1031–48. 10.4054/DemRes.2017.37.33 29551951 PMC5848096

[2] Moore M. The global dimensions of public health preparedness and implications for US action. Am J Public Health. (2012) 102:e1–7. 10.2105/AJPH.2011.300644 22515870 PMC3483965

[3] Fleckman J Dal Corso M Ramirez S Begalieva M Johnson C. Intercultural competency in public health: a call for action to incorporate training into public health education. Front Public Health. (2015) 3:210. 10.3389/fpubh.2015.00210 26389109 PMC4556984

[4] Falconer C Park M Croker H Kessel A Saxena S Viner R . Can the relationship between ethnicity and obesity-related behaviours among school-aged children be explained by deprivation? A cross-sectional study. BMJ Open. (2014) 4:e003949. 10.1136/bmjopen-2013-003949 24413346 PMC3902524

[5] Moolenaar R,. CDC Health Disparities Inequalities Report - United States, 2013. Centers for Disease Control Prevention. (2013). p. 184. Available online at: https://www.cdc.gov/mmwr/pdf/other/su6203.pdf (accessed May 11, 2022).

[6] Deardorff DK. Identification and assessment of intercultural competence as a student outcome of internationalization. J Stud Int Educ. (2006) 10:241–66. 10.1177/1028315306287002

[7] Bennett J. Transformative training: designing programs for culture learning. Contemporary Leadership and Intercultural Competence: Exploring the Cross-Cultural Dynamics within Organizations. SAGE Publications (2009). p. 95–110.

[8] Christie M Carey M Robertson A Grainger P. Putting transformative learning theory into practice. Aust J Adult Learn. (2015) 55:9–30.

[9] Tarchi C Surian A. Promoting intercultural competence in study abroad students. Eur J Psychol Educ. (2022) 37:123–40. 10.1007/s10212-021-00554-0

[10] NAFSA. Association of International Educators. Trends in U.S. Study Abroad. NAFSA. (2020). Available online at: https://www.nafsa.org/policy-and-advocacy/ policy-resources/trends-us-study-abroad (accessed June 1, 2022).

[11] Appiah-Kubi P Annan E. A Review of a collaborative online international learning. Int J Eng Pedagogy. (2020) 10:109–24. 10.3991/ijep.v10i1.11678

[12] Starke-Meyerring D. Globally networked learning environments: reshaping the intersections of globalization and E-learning in higher education. E-Learn Digit Media. (2010) 7:127–32. 10.2304/elea.2010.7.2.27

[13] Karl K Peluchette J Aghakhani N. Virtual work meetings during the COVID-19 pandemic: the good, bad, and ugly. Small Group Res. (2022) 53:343–65. 10.1177/10464964211015286 PMC8165498 38603094

[14] O'Dowd R. From telecollaboration to virtual exchange: state-of-the-art and the role of UNICollaboration in moving forward. J Virtual Exch. (2018) 1:1–23. 10.14705/rpnet.2018.jve.1

[15] Mezirow J. An overview on transformative learning. In: Lifelong Learning: Concepts and Contexts. London: Routledge. (2006) p. 40–54.

[16] Mezirow J. Contemporary paradigms of learning. Adult Educ Q. (1996) 46:158–72. 10.1177/074171369604600303

[17] MacNab B Worthley R. Stereotype awareness development and effective cross-cultural management: an experiential approach. Int J Cross Cult Manag. (2013) 13:65–87. 10.1177/1470595812452635

[18] Ambrose M Murray L Handoyo N Tunggal D Cooling N. Learning global health: a pilot study of an online collaborative intercultural peer group activity involving medical students in Australia and Indonesia. BMC Med Educ. (2017) 17:10. 10.1186/s12909-016-0851-6 28086875 PMC5237179

[19] Niitsu K Kondo A Hua J Dyba N. A case report of collaborative online international learning in nursing and health studies between the United States and Japan. Nurs Educ Perspect. (2022). 10.1097/01.NEP.0000000000000974 35420569

[20] Wood E Collins S Mueller S Stetten N El-Shokry M. Transforming perspectives through virtual exchange: a US-Egypt partnership part 1. Front Public Health. (2022) 10. 10.3389/fpubh.2022.877547 35655459 PMC9152246

[21] Bowen K Barry M Jowell A Maddah D Alami N. Virtual exchange in global health: an innovative educational approach to foster socially responsible overseas collaboration. Int J Educ Technol High Educ. (2021) 18:32. 10.1186/s41239-021-00266-x 34778528 PMC8189728

[22] Collins S Mueller S Wood E Stetten N. Transforming perspectives through virtual exchange: a US-Egypt partnership part 2. Front Public Health. (2022) 10:880638. 10.3389/fpubh.2022.880638 35677760 PMC9168535

[23] Jamshed S. Qualitative research method-interviewing and observation. J Basic Clin Pharm. (2014) 5:87–8. 10.4103/0976-0105.141942 25316987 PMC4194943

[24] Ravitch S Carl N. Qualitative Research: Bridging the Conceptual, Theoretical, and Methodological. Sage Publications (2019).

[25] Husband G. Ethical data collection and recognizing the impact of semi-structured interviews on research respondents. J Educ Sci. (2020) 10:206. 10.3390/educsci10080206

[26] Hussein A. The use of triangulation in social sciences research: can qualitative and quantitative methods be combined? J Comp Soc Work. (2009) 4:106–17. 10.31265/jcsw.v4i1.48

[27] AlYahmady H Alabri S. Using Nvivo for data analysis in qualitative research. Int J Interdiscip Educ. (2013) 1:1–6. 10.12816/000291425606106

[28] Mayring P. Qualitative content analysis. Forum Qual Soc Res. (2000) 1. 10.17169/fqs-1.2.1089

[29] Mayring P. Qualitative content analysis: theoretical foundation, basic procedures, and software solution. Klagenfurt (2014).

[30] Eddy S Hogan K. Getting under the hood: how and for whom does increasing course structure work? CBE Life Sci Educ. (2014) 13:453–68. 10.1187/cbe.14-03-0050 25185229 PMC4152207

[31] Istrate A. Culture shock of studying abroad - new trends for the development of intercultural skills. Romanian Econ Bus Rev. (2018) 13:22–7.

[32] Mezirow J Marsick V. Education for perspective transformation. Women's re-entry programs in community colleges. New York, NY: Columbia University (1978).

[33] DeAngelis L. Enabling the exploration of disorienting dilemma in the classroom. J Educ. (2021) 202:585–600. 10.1177/0022057421991865

[34] Sinulingga A Nasir P Trisni S. Vlog as learning instrument in introduction to international relations to improve student learning outcomes. EUDL. (2019). 10.4108/eai.11-9-2019.2298449

[35] Pillay S James R. Examining intercultural competency through social exchange theory. Int J Teach Learn High Educ. (2015) 27:320–9.

[36] Tompkins A Cook T Miller E LePeau L. Gender influences on students' study abroad participation and intercultural competence. J Stud Aff Res Pract. (2017) 54:204–16. 10.1080/19496591.2017.1284671

[37] Di Pietro G. Changes in the study abroad gender gap: a European cross-country analysis. High Educ Q. (2022) 76:436–59. 10.1111/hequ.12316

[38] Irving CJ English LM. Transformative learning with women: a critical review proposing linkages for the personal and political spheres. In: Adult Education Research Conference. Toronto, ON (2011).

[39] Yue Y Lu J. International students' motivation to study abroad: an empirical study based on expectancy-value theory and self-determination theory. Front Psychol. (2022) 13:841122. 10.3389/fpsyg.2022.841122 35401387 PMC8984802

[40] Hennink M Kaiser B. Sample sizes for saturation in qualitative research: a systematic review of empirical tests. Soc Sci Med. (2021) 292:114523. 10.1016/j.socscimed.2021.114523 34785096

[41] O'Connor C Joffe H. Intercoder reliability in qualitative research: debates and practical guidelines. Int J Qual Meth. (2020) 19. 10.1177/1609406919899220

