# Supplementary material for: Global health-based virtual exchange to improve intercultural competency in students: Long-lasting impacts and areas for improvement
Source: Front Public Health. 2022 Nov 14;10:1044487. doi: 10.3389/fpubh.2022.1044487 (PMC9702054; doi:10.3389/fpubh.2022.1044487)
Supplement: Supplementary file 1 [file Table_1.DOCX]

**Appendix A: Post Virtual Exchange Interview Questions**

1. Before the virtual exchange in 2021, what were your interactions with individuals outside your home country?
2. Since the virtual exchange in 2021, how have those interactions changed (if at all)?
3. Can you describe ways that you’ve been able to integrate any *skills* or *knowledge* gained from the virtual exchange into other courses, your daily life, or other relevant areas? (e.g. communication, listening, self-awareness, problem-solving)
4. Have you changed any *behaviors* as a result of the virtual exchange?
5. What aspect(s) of the virtual exchange did you enjoy most?
6. Within the Global Public Health course, you had several reflections around the virtual exchange experience. Reflecting now, in one word, how would you describe your experience?
